# Who benefits most from studying abroad? A conceptual and empirical overview

**DOI:** 10.1007/s10734-021-00760-1

**Published:** 2021-11-09

**Authors:** Nicolai Netz

**Affiliations:** German Centre for Higher Education Research and Science Studies (DZHW), Lange Laube 12, 30159 Hannover, Germany

**Keywords:** International student mobility, study abroad, personality, labour market outcomes, effect heterogeneity

## Abstract

This editorial to the special issue on heterogeneous effects of studying abroad starts with a review of studies on the determinants and individual-level effects of studying abroad. On that basis, it illustrates the necessity to place more emphasis on effect heterogeneity in research on international student mobility. It then develops a typology of heterogeneous effects of studying abroad, which shall function as an agenda for future research in the field. Thereafter, the editorial introduces the contributions to the special issue. It concludes by summarising major findings and directions for future research.

## Rationale of the special issue

In the last decades, the facilitation of international student mobility (ISM) has been a key action line of European higher education policy (Ferencz & Wächter, 2012). Since the 1950s, ISM has been promoted as a means to generate societal benefits through knowledge exchange, social cohesion, and economic prosperity (Baron, 1993). Since the 2009 Leuven Conference of the European ministers responsible for higher education, policy-makers have additionally emphasised the individual benefits of studying abroad for the mobile students (Ministerial Conference, 2009, 2012).^1^

Along with this development, both policy-makers and scholars have become increasingly interested in who gets access to the benefits of studying abroad. From a variety of disciplinary perspectives—including psychology, educational sciences, economics, and sociology—it matters which factors influence access to studying abroad, and how studying abroad affects individual life courses. In recent years, research has made great progress in answering these questions.

On the one hand, various studies have enhanced our understanding of the factors that influence study abroad participation. These studies have shown, for instance, that the likelihood of studying abroad depends on students’ personality traits (e.g. Bakalis & Joiner, 2004; Zimmermann & Neyer, 2013), beliefs, attitudes, norms, and corresponding benefit expectations (e.g. Petzold & Moog, 2018; Presley et al., 2010; Sánchez et al., 2006; Schnusenberg et al., 2012), socio-demographic features (for an overview, see Netz et al., 2020), such as their gender (e.g. Böttcher et al., 2016; Cordua & Netz, 2021; Salisbury et al., 2010; Van Mol, 2021), age (e.g. Messer & Wolter, 2007; Netz, 2015), ethnicity (e.g. Netz & Sarcletti, 2021; Pungas et al., 2015; Simon & Ainsworth, 2012), and social origin (e.g. Di Pietro, 2020; Lingo, 2019; Netz & Finger, 2016; Waters & Brooks, 2010), previous experience with spatial mobility (e.g. Carlson, 2013; Lörz et al., 2016), academic performance in school and higher education (e.g. Favero & Fucci, 2017; Wiers-Jenssen, 2011; Wiers-Jenssen & Try, 2005), and literacy, numeracy, technical, and foreign language skills (e.g. Di Pietro & Page, 2008; Kommers, 2020). Furthermore, various contextual factors shape students’ opportunities to study abroad. These factors include the attitudes, expectations, and resources of students’ parents (e.g. Bodycott, 2009; Brux & Fry, 2010; Hurst, 2019; Pimpa, 2003) and peers (e.g. Brooks & Waters, 2010; Van Mol & Timmerman, 2014), the support of faculty members (e.g. Paus & Robinson, 2008), students’ field of study (e.g. Iriondo, 2020; Schmidt & Pardo, 2017; Schnepf & Colagrossi, 2020), the design of study programmes (e.g. Perna et al., 2015), the availability of institutional or state funding (e.g. Kramer & Wu, 2021; Whatley, 2017), the economic wealth of countries, and the quality of national higher education systems (e.g. Beine et al., 2014; Rodríguez et al., 2011; Vögtle & Windzio, 2016).

On the other hand, impact evaluations have shown that studying abroad can influence various domains of students’ life courses. For instance, they have illustrated that studying abroad can affect students’ personality development (e.g. Niehoff et al., 2017; Richter et al., 2021; Zimmermann et al., 2021), identity (e.g. King & Ruiz-Gelices, 2003; Sigalas, 2010; Van Mol, 2013), language proficiency (e.g. Brecht et al., 1993; Jackson et al., 2020; Magnan & Back, 2007), multi- or intercultural sensitivity and competences (e.g. Anderson et al., 2006; Clarke et al., 2009; Williams, 2005; Wolff & Borzikowsky, 2018), self-efficacy (e.g. Milstein, 2005; Nguyen et al., 2018; Petersdotter et al., 2017), and academic development and achievement (e.g. Cardwell, 2020; McKeown et al., 2020; Nerlich, 2021; Whatley & Canché, 2021). In recent years, in particular, various studies have also examined the effects of studying abroad on graduates’ labour market outcomes (for an overview, see Netz & Cordua, 2021; Roy et al., 2019; Waibel et al., 2017; Wiers-Jenssen et al., 2020). Among other things, scholars have assessed the effects of studying abroad on the job search duration and the likelihood of employment (e.g. Di Pietro, 2015; Liwiński, 2019a; Petzold, 2017a), skills mismatch (e.g. Wiers-Jenssen & Try, 2005), involvement in international job tasks (e.g. Teichler, 2011; Wiers-Jenssen, 2008), international labour market migration (e.g. Di Pietro, 2012; Parey & Waldinger, 2011), the occupational status (e.g. Waibel et al., 2018), and wages (e.g. Jacob et al., 2019; Kratz & Netz, 2018).

This short literature review illustrates that existing research already provides a comprehensive overview of the determinants and individual-level effects of studying abroad. Yet, it has not sufficiently acknowledged a simple possibility: It is unlikely that all individuals benefit from studying abroad to the same extent. While several studies have performed sensitivity analyses to ensure the robustness of their results across groups of students, educational, employment, and living contexts, as well as types of stays abroad, only a few studies have explicitly focused on heterogeneity in the effects of studying abroad. Mostly, existing studies have concentrated on quantifying an average effect for all individuals in their respective population sample (as becomes evident in several literature reviews: Netz & Cordua, 2021; Roy et al., 2019; Waibel et al., 2017).

However, shifting the focus on effect heterogeneity is beneficial for various reasons—which is already widely acknowledged in the broader literature on returns to higher education (for examples, see Bauldry, 2014; Brand & Xie, 2010; Triventi, 2013; Walker, 2020). As the next section demonstrates, this focus is often a prerequisite for adequately testing specific theoretical assumptions. For instance, assumptions about group differences in individual behaviour and in the returns to education are at the heart of theoretical models deriving from social stratification research.

Explicitly modelling effect heterogeneity can also be imperative methodologically (Breen et al., 2015; Elwert & Winship, 2010). Especially when examining diverse samples of students, the proper specification of an effect of studying abroad usually requires scholars to capture differential selection, that is, individual or group-specific patterns of study abroad participation. Additionally, they need to capture the variables or types of stays abroad across which effects are assumed to exhibit the most substantial variation. In cases where the true effects of studying abroad are likely to differ notably across individuals, groups, or types of stays abroad, one may also question the validity of average effects for entire population samples and of broad summary measures of ISM. Hence, it is both theoretically and methodologically useful to address the question of who benefits most from studying abroad.

Last but not least, answering this question is crucial from a policy perspective. Not only does this create the basis for assessing the political promise that studying abroad yields individual benefits. It also helps answer the question of whether—or rather under which circumstances—the often costly ISM policies pay off. More knowledge about group-specific patterns of selection into ISM could help policy-makers reduce crowding-out effects. More knowledge about heterogeneous returns could ease targeted student support and compensatory measures. Such interventions could increase the efficiency of policy interventions and counteract the often-observed generation of social inequalities in the context of ISM.

## Heterogeneous effects of studying abroad: a typology for future research

Following the methodological literature in the social sciences (e.g. Breen et al., 2015; Carneiro et al., 2011; Elwert & Winship, 2010; Xie et al., 2012), we can conceptually distinguish different types of effect heterogeneity. In a first step, we can differentiate between heterogeneous treatment effects and treatment heterogeneity. A heterogeneous treatment effect arises if the outcome of a specific treatment—that is, an intervention or social phenomenon of interest—varies depending on the values of a third, moderating variable. In contrast, treatment heterogeneity describes the case that different treatments are under examination.

**Fig. 1:**
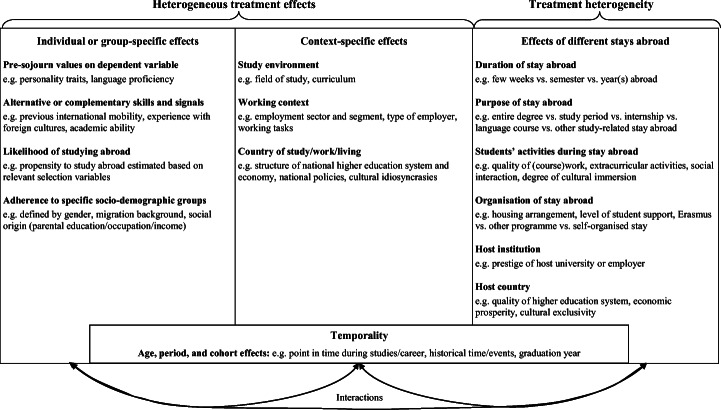
A typology of heterogeneous effects of studying abroad

In research on the outcomes of studying abroad, it is difficult to neatly separate these two types of effect heterogeneity. Because two individuals are unlikely to complete the exact same type of stay abroad in practice, examining heterogeneous treatment effects will usually capture some degree of treatment heterogeneity—which is a problem that might generally not be considered enough in research on the outcomes of social phenomena. Still, applying the insights of the mentioned methodological literature and of different disciplinary approaches enables the development of an entire agenda for future research in the field (see Fig. 1).^2^

To begin with, the effect of studying abroad may be heterogeneous across individuals and groups. First of all, the pre-sojourn values on a dependent variable shape students’ potential to benefit from studying abroad. This perspective is particularly relevant for psychologists and educational scientists, who frequently capture their outcomes of interest using Likert scales. For instance, a very high pre-sojourn conscientiousness naturally limits students to indicate further personality development through studying abroad on a 5-point scale (Niehoff et al., 2017). Vice versa, this does not always imply that students with the lowest pre-sojourn values benefit most from studying abroad. With regard to language acquisition, for example, the potential to benefit from studying abroad seems to be limited for students who lack a linguistic basis to build upon (Magnan & Back, 2007). Thus, students with intermediate values on the examined dependent variables might in many respects be in a good position to benefit from studying abroad.

Relatedly, individuals’ alternative or complementary skills and signals may govern their potential to benefit from studying abroad. For instance, studying abroad could be less beneficial for students who have previously received similar treatments, such as international experience during school or higher education, or home-country experience with foreign cultures (Nguyen et al., 2018). In such cases, the marginal utility of additional international mobility could be decreasing. It is equally possible that sojourns abroad after graduation eclipse the signalling value of study-related stays abroad. Study abroad experience might also substitute other skills or signals. For example, students conveying negative signals, such as poor grades, might compensate their disadvantage through study abroad experience, and thus benefit more from studying abroad than students with good grades. This hypothesis, however, is not supported by initial evidence (Petzold, 2017b). Theoretically, study abroad experience might also reinforce the signalling value of other personal features, and vice versa.

The effects of studying abroad may also vary depending on the likelihood of studying abroad. As the literature on economic returns to studying (abroad) illustrates, there are conflicting hypotheses in this regard: From a classical economic standpoint, the rationally acting and utility-maximising homo oeconomicus should invest in those educational options that are most likely to increase lifetime earnings. Therefore, those individuals who are most likely to study (abroad) should also benefit most from it (Willis & Rosen, 1979). In contrast, the sociological perspective highlights that social norms and opportunity structures influence the likelihood of studying (abroad) as much as rational cost-benefit considerations do (Brand & Xie, 2010). Moreover, contrary to individuals with a low likelihood of studying (abroad), individuals with a high likelihood of studying (abroad) might have good job prospects even if they do not study (abroad). In support of the sociological perspective, existing evidence suggests that students with a lower propensity to study abroad are more likely to benefit from it regarding their job prospects (Waibel et al., 2018, 2020).

From a classical sociological standpoint, it is also relevant to explicitly analyse differences in the effect of studying abroad depending on students’ adherence to specific socio-demographic groups, as defined by ascribed characteristics such as their gender, migration background, and social origin. As shall be illustrated regarding social origin, this social stratification perspective also allows for competing scenarios: On the one hand, students from a high social origin could benefit more from studying abroad. They tend to be better equipped with material and cultural resources allowing them to profit from education (Savage & Egerton, 1997). Moreover, their habitus and capital endowments may allow them to better valorise their experiences and credentials in the labour market (Laurison & Friedman, 2016). On the other hand, students from a low social origin could benefit more. Considering that they are less likely to gain the skills and signals acquirable through studying abroad during their earlier life course, studying abroad could induce a compensatory levelling process (Schafer et al., 2013). Furthermore, students from a low social origin may be positively selected in terms of motivation and productivity characteristics, which could positively influence their likelihood of studying abroad and their later potential to capitalise on it. As they usually have to overcome higher financial and social burdens, they might solely decide to study abroad if they are strongly convinced of reaping its benefits (Waibel et al., 2020).^3^

The effects of studying abroad are also likely to be context-specific. This means that stays abroad tend to result in different outcomes depending on the surroundings in which individuals live, study, or work. For example, the value of stays abroad will likely vary depending on students’ field of study (Nerlich, 2021). Studying abroad may be more relevant for academic development in modern languages and cultural sciences than, for instance, in chemistry. Its value may even vary depending on specific curricula within fields of study.

There is further reason to assume that graduates’ working contexts moderate the effects of studying abroad. The employment sector may moderate the effects of studying abroad in that private companies tend to remunerate study abroad experience more than public authorities (Wiers-Jenssen, 2011). Public-sector wage schemes are usually more rigid and can less flexibly reward additional assets such as study abroad experience. Its value may also vary across labour market segments: The value of study abroad experience may be higher in vocationally unspecific segments, in which graduates of fields such as the humanities, social sciences, and economics tend to work, than in vocationally specific segments, in which graduates of fields such as medicine and teaching tend to work (Kratz & Netz, 2018; Waibel et al., 2018). The reason could be that the rules of career success are more strictly regulated in vocationally specific segments, so that add-on signals are less valuable. Moreover, study abroad experience seems to pay off particularly when graduates work for multinational employers (Petzold, 2017a; Wiers-Jenssen & Try, 2005). Eventually, the value of study abroad experience may largely depend on the working tasks that graduates complete on a daily basis.

The effects of studying abroad may further vary across the country of study, work, and living. To some extent, national differences regarding the already discussed features of study environments and working contexts may explain cross-country variation. Beyond that, there may be differences in the extent to which national higher education systems reward study abroad experience. So far, however, most internationally comparative studies have focused on differences in the labour market effects of studying abroad depending on the structure of national economies. These studies suggest that labour market returns to studying abroad tend to be highest in Southern and Eastern European countries, moderate in Central European countries, and smallest or even non-existent in Northern European countries (Humburg & van der Velden, 2015; Jacob et al., 2019; Rodrigues, 2013; Teichler, 2011; Van Mol, 2017). Adding to country-specific explanations (e.g. Van Mol, 2017), Jacob et al. (2019) suggest that “returns to international study experience in terms of hourly wage and class position [are] larger in countries with poorer university quality, lower international trade volume, higher graduate unemployment, and with relatively few students going abroad” (p. 500).^4^

Besides structural features of higher education systems and economies, national policies may influence the effects of studying abroad, e.g. through programmes trying to attract internationally experienced graduates. Furthermore, various cultural idiosyncrasies—as defined e.g. by the national social system, prevalent religion and gender roles, openness to foreigners, degree of urbanisation, and official language(s)—might moderate the effects of studying abroad. In these respects, internationally comparative research is still in its infancy.

Regarding treatment heterogeneity, various facets of stays abroad are relevant from both theoretical and policy perspectives. The first facet is the duration of the stay abroad. Arguably, effects of studying abroad are—on average—less likely to manifest following very short stays of just a few days or weeks than following longer stays of several months or years (Dwyer, 2004). Some authors presume that the effect of studying abroad rises linearly with the time spent abroad. For example, Medina-López-Portillo (2004) “suggests that the longer the program, the more interculturally sensitive students are likely to become” (p. 185). It is equally possible that the learning curve and thus the marginal utility decrease with the time spent abroad, so that the relationship would follow a logarithmic pattern. Some evidence on the labour market effects of studying abroad is even in line with an inverted U-shape pattern, suggesting that the signalling value of stays abroad may first increase but then decrease again with rising duration. For instance, Rodrigues (2013) reports that studying abroad for three to 12 months yields a moderate wage premium, while studying abroad for less than three or more than 12 months yields no significant wage returns. Yet other studies report no effect heterogeneity depending on the time spent abroad. For instance, Schmidt and Pardo (2017) find no significant differences in the wage effects of 3-to-4 weeks as opposed to full-terms abroad.

The duration closely relates to the purpose of a stay abroad, which emphasises its function for competence development. For example, entire degrees and study periods abroad are likely to foster academic and generic intercultural skills, internships should help students acquire human capital that is particularly relevant professionally, and language courses may be most effective in improving language proficiency. Research comparing the effects of study periods and internships abroad concludes that internships abroad pay off slightly more in the labour market (Kratz & Netz, 2018; Van Mol, 2017).^5^ A specific discussion revolves around the question of whether studying abroad entirely or partly is most beneficial. Evidence from Norway suggests that wage returns are higher for entire degrees than for study periods completed abroad (Wiers-Jenssen, 2011; Wiers-Jenssen & Try, 2005). In contrast, evidence from several (other) European countries suggests that employers prefer graduates who partly studied abroad over those who entirely studied abroad (Humburg & van der Velden, 2015). Ultimately, the extent to which graduates need general and country-specific human capital for their daily working life will be decisive in this respect.

An even more explicit focus on students’ actual activities is beneficial as well. Not least due to lacking standard criteria for evaluating the quality of stays abroad and of corresponding data, (quantitative) scholars have so far mostly treated stays abroad as black boxes concerning students’ activities. Logically, the quality of the coursework or work assignments matters. High-quality courses and ambitious assignments will likely influence the development of academic and professional skills more positively than sojourns that largely resemble touristic stays. Besides academic and professional activities, extracurricular activities may have a substantial bearing on the outcomes of studying abroad (Gozik & Oguro, 2020). In academic, professional, and extracurricular terms, students’ social contacts and the degree of immersion in their host culture also seem to play a vital role. For instance, establishing new relationships abroad is an essential catalyst for the positive effects of studying abroad on personality development (Zimmermann & Neyer, 2013). Similarly, intense interaction with host-country nationals is particularly important for improving oral foreign language proficiency (Engle & Engle, 2004; Jackson et al., 2020; Magnan & Back, 2007).

In this respect, the organisation of stays abroad comes into play. For instance, students’ housing arrangement—that is, whether they live in a host family, student residence, or off-campus apartment either with co-nationals, other non-nationals, host-country nationals, mixed groups, or alone—has received considerable attention in the study abroad literature. Regarding gains in language proficiency and other intercultural skills, however, the housing arrangement alone does not seem to be very predictive (Gozik & Oguro, 2020; Jackson et al., 2020). Rather, the previously discussed activities seem to matter. Moreover, a solid but not excessive level of student support, including pre-sojourn administrative and academic preparation, organisational support in the host country, post-sojourn follow-up reflection, and credit recognition can help students reap the benefits of studying abroad (Gozik & Oguro, 2020; Norris & Dwyer, 2005). Participation in specific study abroad programmes, as opposed to self-organised stays, may also influence the outcomes of studying abroad. Different programmes and self-organised stays abroad could either reflect the previously discussed types of treatment heterogeneity or have an unequal signalling value due to more or less restrictive or non-existent eligibility criteria.^6^

The effects of studying abroad will arguably also depend on the host institution. Host universities and employers offering high-quality education, support, or working conditions should bring about better outcomes than institutions offering poor opportunity structures. In line with this view, there is evidence that European employers regard the prestige of graduates’ (host) universities during hiring processes as a signal of graduates’ level of skill acquisition (Humburg & van der Velden, 2015).

If employers cannot appraise the quality of graduates’ host institution, they may also draw on their own assumptions or factual information about the host country. For instance, stays in countries with effective higher education systems may signal high-quality learning experiences. Stays in countries with prosperous economies may signal the acquisition of professionally relevant skills. And stays in culturally exclusive countries may enable social distinction. Although only loosely linked to these theoretical thoughts, there is initial evidence on the labour market effects of sojourning in specific host countries: Examining graduates from institutions in Spain, Iriondo (2020) reports that wage returns to participation in the Erasmus programme are highest for stays in Germany, followed by stays in France, the Nordic countries, and the UK. Stays in countries such as Italy and Portugal do not seem to yield significant wage returns. Concentrating on returns to language acquisition rather than stays in specific host countries, Sorrenti (2017) reports that proficiency in German yields the highest wage returns for graduates from Italy, followed by proficiency in English, French, and Spanish. While there is some overlap between these findings, they also suggest that the value of stays in specific countries varies depending on graduates’ home country—and arguably also depending on various other factors, including the specific career that graduates intend to pursue.

Finally, temporality matters for analysing the outcomes of studying abroad. Methodologically, it is useful to differentiate age, period, and cohort effects (Winship & Harding, 2008). Age effects could result from the timing at which a stay abroad is completed. For instance, a stay abroad close to graduation might have stronger effects on the likelihood of employment than a stay abroad shortly after entering higher education. The former could help students broaden their professional networks and gain valuable information for their upcoming job search. In turn, an early stay abroad might have more substantial effects on academic development. Moreover, what matters is the point in graduates’ careers when we measure the outcomes of studying abroad. Existing evidence suggests that specific labour market effects of studying abroad may take several years to unfold (Netz & Cordua, 2021). A reason could be that the competences acquired through studying abroad cannot be applied immediately in many labour market entry positions.

Period effects would find their expression in a changing value of study abroad experience over time. Teichler and Janson (2007) suggest that the self-perceived professional value of Erasmus study abroad experience may have decreased between the late 1980s and 2005 with the increasing share of students studying abroad. While the scarcity value of study abroad experience has certainly decreased, it is equally possible that the skills acquired through studying abroad have become more relevant in continuously globalising labour markets.

Cohort effects are characterised by common events experienced by specific groups. For instance, the 2020 and 2021 graduation cohorts may not have been able to readily capitalise on possible study abroad experience because of hiring freezes and limited international cooperation in the wake of the COVID-19 pandemic. This may translate to long-term disadvantages (scarring effects) for these cohorts.

As already indicated, different types of effect heterogeneity may interact—or rather define an outcome in conjunction. For instance, we might observe different effects of studying abroad across social groups partly because different groups are more or less likely to work in specific labour market segments, where study abroad experience is either more or less remunerated. This pattern could also result from different social groups completing different stays abroad. Whether different study abroad treatments produce divergent effects may depend on the country of work/living. Finally, as time and space features are not separable, the discussed age, period, and cohort effects will always be defined by individual or group-specific effects, context-specific effects, and treatment heterogeneity. Clearly, it is difficult to empirically disentangle different types of effect heterogeneity using currently available data and methods. Still, their conceptual differentiation is vital for appropriate hypothesis testing and for pinpointing effective policy recommendations.

## Articles of the special issue

The articles of this special issue engage with the developed research agenda. In doing so, they each contribute a unique analytical perspective by accentuating specific disciplinary angles, corresponding theoretical and methodological approaches, country contexts, outcomes of studying abroad, and types of effect heterogeneity.

The articles have their roots in psychology, economics, and sociology. Relatedly, they use diverse theoretical approaches (theories of personality traits, experiential learning, rational choice, human capital, signalling, statistical discrimination, social capital, and social stratification) and statistical methods (linear and multinomial logistic regressions, latent change models, multilevel models, growth curve models, and propensity score matching). They cover Anglo-Saxon, Continental and Southern European, and Scandinavian countries (UK, Germany, The Netherlands, Italy, and Norway). They focus on different outcomes of studying abroad (multicultural self-efficacy, metacognitive intercultural competence, intergroup anxiety, uptake of postgraduate education, job search duration, likelihood of employment, skills mismatch, and labour income). Thereby, they also explore the effects of studying abroad in different life course stages (during studies, the transition from higher education to work, and the early professional career). Finally, they consider a variety of the above-mentioned types of effect heterogeneity. These include individual or group-specific effects (contingent on pre-mobility values of specific dependent variables, alternative skills and signals, the likelihood of studying abroad, and the adherence to specific socio-demographic groups), context-specific effects (as defined by the study environment, working context, and country of work), treatment heterogeneity (depending on the purpose, organisation, and host country of stays abroad), and aspects of temporality (point during studies when a stay abroad was completed, point in career when its effect was measured, and graduation year).

The articles also have commonalities: In response to repeated calls for better approximations of causal effects of studying (e.g. Netz & Cordua, 2021; Waibel et al., 2017; Wiers-Jenssen et al., 2020), all articles employ statistical techniques that can reduce the bias resulting from the selective participation in ISM. Thereby, they also contribute to integrating the still often disconnected research streams on the determinants and on the effects of studying abroad. Moreover, they either use large-scale and mostly nationally representative observational data or experimental data to ensure the validity of the generated results. Some studies examine the same countries, types of stays abroad, outcomes, or types of effect heterogeneity. This allows for rough comparisons of their results.

In the first article, *Julia Zimmermann*, *Henriette Greischel*, and *Kathrin Jonkmann* (2020) examine the influence of studying abroad on different facets of multicultural effectiveness. Based on psychological theories of personality traits and experiential learning, they reason that studying abroad should increase multicultural self-efficacy as well as metacognitive intercultural competence and decrease intergroup anxiety. They also assume that these effects vary depending on selected socio-demographic characteristics and students’ previous international mobility. They test their hypotheses based on a countrywide purposive sample of students at higher education institutions in Germany, whom they surveyed three times during their studies. Using latent change models, they find evidence supporting their theoretical assumptions: Studying abroad slightly increases self-perceived multicultural self-efficacy and metacognitive intercultural competence. Moreover, it slightly lowers intergroup anxiety. Importantly, these developmental patterns do not vary depending on students’ socio-demographics—as defined by their gender, age, migration background, and parents’ professional qualification. However, students benefit most from studying abroad regarding the development of multicultural effectiveness when they are internationally mobile for the first time.

In the second article, *Knut* *Petzold* (2020) addresses the relevance of study abroad experience during hiring processes. Following economic theories of human capital, job market signalling, and statistical discrimination, he examines how the importance that human resource managers attach to studying abroad varies depending on the purpose and timing of stays abroad, graduates’ socio-demographic features, their other human capital characteristics, and the (inter)national orientation of employers. He bases his analysis on a factorial survey experiment administered to a purposive sample of German employers. Estimating multilevel models, he finds suggestive evidence that employers consider internships the most valuable (arguably because they generate the most specific human capital), followed by study periods and non-educational private stays abroad. Graduates with a migration background benefit less from study periods and private stays abroad than graduates without such a background, possibly because a migration background already signals transnational human capital. Also, Master graduates benefit less from study periods and internships abroad because they may already have more general and specific human capital than Bachelor graduates. Finally, employers value study abroad experience (insignificantly) more if they have a foreign branch, which could indicate a relatively higher value of transnational human capital for multinational employers.

In the third article, *Jannecke Wiers-Jenssen* and *Liv Anne Støren* (2020) explore whether studying abroad affects the risk of unemployment and skills mismatch about six months after graduation. Following theories of human capital and signalling, they hypothesise that this risk differs depending on graduates’ socio-demographics and working context. They test their hypotheses based on data from the Norwegian graduate survey. These data cover six graduation cohorts, who completed their studies between 2007 and 2017. Their multinomial logistic regressions show that most differentiated graduate groups do not differ significantly in their risk of unemployment and skills mismatch depending on whether they have studied abroad. However, they find that studying abroad reduces this risk among graduates of business and administration, who tend to work in the private sector. They conclude that their results contradict the hypothesis that study abroad experience pays off mainly among graduates of vocationally unspecific fields. Furthermore, they find that studying abroad reduces the risk of unemployment and skills mismatch particularly among graduates with high intake grades. They do not observe effect heterogeneity depending on the social origin or migration background. Therefore, they conclude that their results also contradict the hypothesis that those less likely to study abroad profit more from it.

In the fourth article, *Christof Van Mol*, *Kim Caarls*, and *Manuel Souto-Otero* (2020) assess the effect of studying abroad on the duration of the transition from higher education to work and on the monthly wage at 1.5 years after graduation. Starting from theoretical thoughts on human capital, signalling, and international prestige hierarchies of higher education systems and labour markets, they look at effect heterogeneity depending on the study level (Bachelor vs. Master), purpose of a stay abroad (study period vs. internship vs. both), and educational and economic features of students’ host countries. They test their hypotheses based on nationally representative graduate survey data from the Netherlands. Using linear regressions, they observe that the examined labour market effects of studying abroad vary slightly across study levels, purposes of stays abroad, and host countries. Against expectations, however, the observed effects and corresponding heterogeneity largely disappear after stricter controls for selection effects through propensity score matching. Also contrary to expectations, sojourns in countries with well-performing higher education systems come along with a longer duration of job search, possibly because students staying in such countries take more time to find jobs matching their high aspirations. Overall, the authors conclude that the well-performing higher education system and labour market in the Netherlands restrict graduates’ potential to further improve their labour market prospects through studying abroad.

In the fifth article, *Béatrice d’Hombres* and *Sylke Schnepf* (2021) examine the effect of studying abroad on the likelihood of postgraduate education and of employment in the first years after graduation. Referring to human capital, signalling, and social capital theories, they compare these labour market effects of studying abroad across countries and socio-economic groups. They draw on large-scale graduate survey data from Italy and the UK to test their hypotheses. In line with theory, their matching analyses indicate that studying abroad correlates with a greater likelihood of postgraduate education among graduates in Italy. They do not observe this link among graduates in the UK. The effect of studying abroad on the likelihood of employment is significantly positive both one and four years after graduation in Italy. In the UK, it is significantly positive six months after graduation and insignificant three years after graduation. Thus, the examined labour market returns to studying abroad are higher in Italy than in the UK. Against expectations, the effects of studying abroad on the likelihood of employment do not differ significantly across socio-economic groups. However, the effect of studying abroad on the likelihood of postgraduate education is larger among graduates from a low socio-economic background than among those with a high socio-economic background in Italy.

In the last article, *Nicolai Netz* and *Michael Grüttner* (2020) provide a sociological analysis of the relationship between studying abroad and the generation of social inequality. Drawing on social stratification theory, they argue that a scenario in which ISM increases social inequality (because graduates from an academic background benefit from cumulative advantages) is as plausible as a scenario in which ISM decreases social inequality (because graduates from a non-academic background benefit from compensatory levelling). Following these thoughts, they test whether the effect of studying abroad on labour income varies across social groups in the German labour market. Their study is based on nationally representative survey data capturing the first ten years of graduates’ careers, which they analyse using propensity score matching and random effects growth curve models. In line with the scenario of cumulative advantage, their results suggest that graduates from an academic background benefit more from studying abroad than graduates from a non-academic background. Considering that students from an academic background are also more likely to study abroad in the first place, they conclude that ISM fosters the reproduction of social inequality. They also find that the estimated returns to studying abroad are highest among those with the lowest propensity to study abroad. However, this pattern seems to be driven by the results for graduates from an academic background.

Taken together, the articles of the special issue provide a comprehensive answer to the question of who benefits most from studying abroad. At the same time, they clearly indicate a need for further research. Some major findings and directions for future research are highlighted in the concluding section.

## Summary and conclusions

It is beyond the scope of this editorial to comprehensively summarise the wealth of empirical evidence that the articles of the special issue provide. However, the following lines highlight a few overarching themes.

To begin with, all contributions to the special issue illustrate that studying abroad has only moderate effects on the examined outcomes—if compared to other critical life events, skills, and signals. They equally demonstrate that the benefits of studying abroad are often confined to specific groups of students and graduates, contexts, and types of stays abroad. Consequently, they justify the initial claim that research on ISM should devote more attention to effect heterogeneity.

Additionally, the articles highlight the importance of adopting a life course perspective. This perspective does not only help scholars trace group-specific patterns of selection into study abroad experience. It also emphasises that specific groups of students may build up cumulative advantages or disadvantages over their life course due to (even minor) heterogeneous effects of studying abroad (Zimmermann et al., 2020). The life course perspective also stresses the importance of other aspects of temporality: Although further research is needed in this respect, there is evidence that the timing of a stay abroad matters (Petzold, 2020; Van Mol et al., 2020). Moreover, the effect of studying abroad seems to vary over graduates’ careers: Country differences notwithstanding, the labour market effects of studying abroad—especially with regard to labour income—seem to be more pronounced a few years after graduation than shortly thereafter (d’Hombres & Schnepf, 2021; Netz & Grüttner, 2020; Van Mol et al., 2020; Wiers-Jenssen & Støren, 2020).

Furthermore, the contributions to the special issue have produced evidence of diminishing marginal returns of gaining additional international experience. For instance, gains in multicultural effectiveness are particularly notable among students without previous sojourns abroad (Zimmermann et al., 2020). Also, graduates who can signal transnational human capital in other ways are less likely to benefit from studying abroad in terms of their probability of being hired (Petzold, 2020).

To further advance our knowledge on (heterogeneous) effects of studying abroad, we need panel data covering longer time frames. These data should ideally describe individuals’ life courses starting at early ages and throughout their entire educational and professional career. Such data would not only allow us to answer questions that are inherently longitudinal in nature, but also to integrate ISM research rooted in different disciplines and research communities. This would enable a shift from multidisciplinary to interdisciplinary research on the effects of studying abroad. For instance, it would be relevant to examine how differential changes in personality traits and intercultural competences due to study abroad experience translate into group-specific labour market outcomes. Answering such questions would also provide more knowledge about the mechanisms that can explain the observed heterogeneity in the effects of studying abroad.

Additionally, long-running panel data would bring about methodological advances: They would enable the application of statistical techniques allowing for better approximations of causal effects of studying abroad. At present, many surveys limit analyses of heterogeneous outcomes of studying abroad because they address individuals only after graduation. This limitation of graduate surveys explains the relative popularity of matching approaches, which cannot capture selection into study abroad experience based on unobserved characteristics. A fruitful complement to the extension of survey data would be the more frequent use of experimental designs in research on ISM.

Besides age effects, period effects and cohort effects warrant further attention in research on ISM. Once the required panel data are available for multiple student and graduate cohorts, scholars could examine whether the effects of studying abroad have changed over time. For instance, we still lack robust analyses testing the hypothesis that the labour market returns to studying abroad have declined over the past decades as a result of ISM becoming less exclusive (see also Waibel et al., 2017).^7^

In line with previous evidence on occupational status benefits of studying abroad (Waibel et al., 2018, 2020), evidence on the wage effects of studying abroad presented in this special issue confirms the tendency that those with a low propensity to study abroad benefit more from studying abroad than those with a high propensity to study abroad (Netz & Grüttner, 2020). However, it is noteworthy that all existing studies refer to graduates in the German labour market. Thus, further evidence is needed from other countries.

The findings are far less straightforward concerning effect heterogeneity depending on the social origin. In Italy, students from lower socio-economic backgrounds benefit more from studying abroad in terms of foreign language acquisition (Sorrenti, 2017) and the likelihood of postgraduate education (d’Hombres & Schnepf, 2021). Regarding the employment likelihood a few years after graduation, analyses of the returns to studying abroad report either no significant group differences (d’Hombres & Schnepf, 2021) or comparatively high returns for graduates from intermediate social backgrounds (Di Pietro, 2015). In Norway, the influence of studying abroad on graduates’ early-career risk of unemployment and skills mismatch does not vary significantly depending on parents’ educational attainment (Wiers-Jenssen & Støren, 2020). Similarly, Zimmermann et al. (2020) do not find significant differences by parents’ professional qualifications in the effect of studying abroad on multicultural effectiveness among students in Germany. However, wage returns to studying abroad are higher among graduates from an academic background in the German labour market (Netz & Grüttner, 2020). In Poland, too, graduates from an academic background benefit most from studying abroad in terms of the employment probability (Liwiński, 2019a).

Concerning the migration background, the effect of studying abroad on multicultural effectiveness does not vary significantly in Germany (Zimmermann et al., 2020). Similarly, the effect of studying abroad on the risk of unemployment and skills mismatch does not vary significantly depending on whether graduates have a migration background in Norway. However, graduates with a migration background seem to benefit slightly less from study periods and private stays abroad regarding the propensity of being hired in Germany (Petzold, 2020). In summary, existing evidence on heterogeneous effects of studying abroad depending on socio-demographics is thus mixed.^8^

Furthermore, there is conflicting evidence regarding the hypothesis that study abroad experience pays off more in vocationally unspecific than in vocationally specific labour market segments. Evidence from Germany concerning the influence of studying abroad on occupational status (Waibel et al., 2018) and on labour income (Kratz & Netz, 2018; Netz & Grüttner, 2020) supports this hypothesis. However, Wiers-Jenssen and Støren (2020) find no evidence of this pattern regarding the risk of unemployment and skills mismatch in Norway.

Further research should address the reasons for the highlighted inconsistencies. One reason could be that studies use different variables to capture ISM, the social and migration backgrounds, the specificity of labour market segments, and the respective outcome variables. Another possible reason is the use of different analytical samples and methods. It is also conceivable that students from specific backgrounds benefit more from studying abroad regarding skill acquisition, but are not able to translate such relative advantages to tangible labour market benefits. Finally, the highlighted inconsistencies could also reflect country differences in how national higher education systems and labour markets moderate the effects of studying abroad.

Analyses of effect heterogeneity depending on the likelihood of studying abroad, socio-demographics, and the fields of study and work are just some examples where high-quality, large-scale, internationally comparable data are dearly needed. To date, research on country differences in the effect of studying abroad is confined to European countries (Humburg & van der Velden, 2015; Jacob et al., 2019; Rodrigues, 2013; Teichler, 2011; Van Mol, 2017). While the contributions to the special issue are not always in line with the pattern that labour market effects of studying abroad are “larger in countries with poorer university quality, lower international trade volume, higher graduate unemployment, and with relatively few students going abroad” (Jacob et al., 2019, p. 500), they align with the geographic pattern that returns tend to be larger in Southern than in Central European countries and smallest in Northern European countries: The contributions report notably positive labour market effects of studying abroad in Italy, moderately positive effects in the UK (d’Hombres & Schnepf, 2021) and Germany (Netz & Grüttner, 2020; Petzold, 2020), and slightly positive or insignificant effects in Norway (Wiers-Jenssen & Støren, 2020) and the Netherlands (Van Mol et al., 2020).

Regarding treatment heterogeneity, the results presented in the special issue (Petzold, 2020) are in line with previous research suggesting that employers place more value on internships abroad than on study periods abroad (Van Mol, 2017). In Germany, this also seems to translate to slightly higher wage effects of internships than of study periods abroad (Kratz & Netz, 2018). In the Netherlands, however, analyses of graduate survey data do not reveal significant differences in this regard (Van Mol et al., 2020). Considering that only a few studies explore this treatment heterogeneity, further research is needed. This claim also applies to heterogeneity depending on the organisation and host country of stays abroad. In this respect, it is important to keep in mind—and certainly also difficult to model with the available sample sizes—that students’ and graduates’ potential to benefit from studying abroad will likely depend on the specific pairing of their home and host countries.

The findings on treatment heterogeneity are probably also highly contingent on the examined dependent variables. Studies differentiating the effects of study periods and internships abroad have focused on labour market effects. Considering that internships are more likely to generate specific, labour market relevant human capital than study periods abroad, it is understandable that employers favour internships over study periods. The picture might look different, for instance, in the expanding line of research examining the effects of studying abroad on academic development and achievement.

Besides this outcome, ISM scholarship could also devote more attention to further dependent variables that have received little attention—in research on studying abroad in general and in research on corresponding effect heterogeneity in particular. For instance, it would be relevant to examine (heterogeneous) effects of studying abroad on relationship stability, health-related quality of life, and life satisfaction.

In summary, this special issue has compiled manifold conceptual angles and empirical findings on heterogeneous effects of studying abroad. It has equally illustrated the ample opportunities to further expand research on ISM through a more explicit focus on effect heterogeneity.^9^ Clearly, the proposed typology of heterogeneous effects of studying abroad has not yet been fully explored empirically. In that sense, we have only just begun to answer the question of who benefits most from studying abroad.

## Footnotes

1 In this special issue, study(ing) abroad denotes study-related stays outside the country where students obtained their higher education entrance qualification or where they first enrolled in higher education. Study(ing) abroad may take the form of entire degrees completed abroad (often called degree mobility or diploma mobility) or of study periods, internships, language courses, and other study-related stays abroad (also referred to as short-term mobility, or as credit mobility if yields study credits). The term study(ing) abroad is often used synonymously to the notion of international student mobility (ISM). Strictly speaking, however, study(ing) abroad accentuates individual action on a micro level, while ISM rather describes cross-border flows of students from a macro perspective.

2 The developed typology does not present a complete list of conceivable types of heterogeneity in the individual-level effects of studying abroad. Rather, it collates the major types that have so far been discussed in the ISM literature. It also intends to inspire thoughts about other types of heterogeneity in the effects of studying abroad. Ultimately, it aims at broadening the ISM research agenda.

3 Clearly, approaches examining effect heterogeneity depending on latent constructs or composite measures, such as the propensity to study abroad, are related to approaches using manifest constructs, such as the social origin being measured by the educational attainment of students' parents. In turn, both analytical perspectives are related to analyses of effect heterogeneity focussing on pre-sojourn values on dependent variables or on other skills and signals. In fact, students with differing propensity to study abroad and social origin will also differ regarding these latter two aspects. However, as has been illustrated, it is legitimate to conceptually differentiate all four perspectives because they accentuate different theoretical assumptions. Moreover, as the contributions to the special issue demonstrate, they require different methodological strategies and may produce different results.

4 From a country-of-origin perspective, the returns to studying abroad can also vary depending on the country where national graduates find work. Among graduates from Poland, for instance, wage returns to studying abroad seem to manifest only among graduates working abroad after graduation, and not among graduates remaining in Poland (Liwiński, 2019b).

5 From a policy perspective, it would also be interesting to examine the outcomes of hybrid types of stays abroad. For instance, do combinations of study periods and internships abroad produce particularly beneficial results?

6 Cammelli et al. (2008) provide descriptive evidence that the early-career wages of Erasmus alumni exceed the wages of graduates who organised their stays abroad differently. In future research, experimental designs—such as vignette studies or field experiments—would offer ample opportunities to provide more robust tests of differences in the signalling value of specific study abroad programmes and of self-organised stays abroad.

7 Combinations of different data sources could also advance research. Examples are the inclusion of survey experiments into student and graduates surveys, and the linkage of survey data with administrative data from higher education institutions, social security offices, and tax bureaus. Such integrated data would provide very robust measures of students’ and graduates’ spatial, educational, and labour market trajectories.

8 Regarding students’ and graduates’ socio-demographics, in particular, further research should better examine the role of policy interventions for the generation of inequalities. With few exceptions (e.g. Kramer & Wu, 2021; Netz & Finger, 2016), it has hardly been examined how policy interventions influence socio-demographic inequalities in study abroad participation. We know even less about how policies may inadvertently promote unequal outcomes of studying abroad, or about effective policies to maximise the benefits of studying abroad for disadvantaged student groups.

9 By drawing on respectively relevant theoretical models, such a focus could certainly also help advance research in other realms of the social sciences.

## References

[CR1] Anderson P, Lawton L, Rexeisen R, Hubbard A (2006). Short-term study abroad and intercultural sensitivity: A pilot study. International Journal of Intercultural Relations.

[CR2] Bakalis S, Joiner T (2004). Participation in tertiary study abroad programs: The role of personality. International Journal of Educational Management.

[CR3] Baron B (1993). The politics of academic mobility in Western Europe. Higher Education Policy.

[CR4] Bauldry S (2014). Conditional health-related benefits of higher education: An assessment of compensatory versus accumulative mechanisms. Social Science & Medicine.

[CR5] Beine M, Noël R, Ragot L (2014). Determinants of the international mobility of students. Economics of Education Review.

[CR6] Bodycott P (2009). Choosing a higher education study abroad destination: What mainland Chinese parents and students rate as important. Journal of Research in International Education.

[CR7] Böttcher L, Araújo N, Nagler J, Mendes J, Helbing D, Herrmann H (2016). Gender gap in the ERASMUS mobility program. PLoS One.

[CR8] Brand J, Xie Y (2010). Who benefits most from college? Evidence for negative selection in heterogeneous economic returns to higher education. American Sociological Review.

[CR9] Brecht, R., Davidson, D., & Ginsberg, R. (1993). *Predictors of foreign language gain during study abroad*. National Foreign Language Center.

[CR10] Breen R, Choi S, Holm A (2015). Heterogeneous causal effects and sample selection bias. Sociological Science.

[CR11] Brooks R, Waters J (2010). Social networks and educational mobility: The experiences of UK students. Globalisation, Societies and Education.

[CR12] Brux J, Fry B (2010). Multicultural students in study abroad: Their interests, their issues, and their constraints. Journal of Studies in International Education.

[CR13] Cammelli, A., Ghiselli, S., & Mignoli, G. (2008). Study experience abroad: Italian graduate characteristics and employment outcomes. In M. Byram & F. Dervin (Eds.), *Students, staff and academic mobility in higher education* (pp. 217–236). Cambridge Scholars Publishing.

[CR14] Cardwell P (2020). Does studying abroad help academic achievement?. European Journal of Higher Education.

[CR15] Carlson S (2013). Becoming a mobile student – A processual perspective on German degree student mobility. Population, Space and Place.

[CR16] Carneiro P, Heckman J, Vytlacil E (2011). Estimating marginal returns to education. American Economic Review.

[CR17] Clarke I, Flaherty T, Wright N, McMillen R (2009). Student intercultural proficiency from study abroad programs. Journal of Marketing Education.

[CR18] Cordua, F., & Netz, N. (2021). Why do women more often intend to study abroad than men? *Higher Education*. 10.1007/s10734-021-00731-6.

[CR19] d’Hombres, B., & Schnepf, S. (2021). International mobility of students in Italy and the UK: Does it pay off and for whom? *Higher Education*. 10.1007/s10734-020-00631-1.

[CR20] Di Pietro G (2012). Does studying abroad cause international labor mobility? Evidence from Italy. Economics Letters.

[CR21] Di Pietro G (2015). Do study abroad programs enhance the employability of graduates?. Education Finance and Policy.

[CR22] Di Pietro G (2020). Changes in socioeconomic inequality in access to study abroad programs: A cross-country analysis. Research in Social Stratification and Mobility.

[CR23] Di Pietro G, Page L (2008). Who studies abroad? Evidence from France and Italy. European Journal of Education.

[CR24] Dwyer, M. (2004). More is better: The impact of study abroad program duration. *Frontiers: The Interdisciplinary Journal of Study Abroad, 10*(1), 151–163. 10.36366/frontiers.v10i1.139.

[CR25] Elwert, F., & Winship, C. (2010). Effect heterogeneity and bias in main-effects-only regression models. In R. Dechter, H. Geffner, & J. Halpern (Eds.), *Heuristics, probability and causality: A tribute to Judea Pearl* (pp. 327–336). College Publications.

[CR26] Engle, L., & Engle, J. (2004). Assessing language acquisition and intercultural sensitivity development in relation to study abroad program design. *Frontiers: The Interdisciplinary Journal of Study Abroad, 10*(1), 219–236. 10.36366/frontiers.v10i1.142.

[CR27] Favero, L., & Fucci, A. (2017). The Erasmus effect on earnings: A panel analysis from Siena. *Quaderni del Dipartimento di Economia Politica e Statistica, 672*, 1–45.

[CR28] Ferencz, I., & Wächter, B. (Eds.). (2012). *European and national policies for academic mobility. Linking rhetoric, practice and mobility trends*. Lemmens.

[CR29] Gozik, N., & Oguro, S. (2020). Program components: (Re)considering the role of individual areas of programming in education abroad. In A. Ogden, B. Streitwieser, & C. Van Mol (Eds.), *Education abroad: Bridging scholarship and practice* (pp. 59–72). Routledge.

[CR30] Humburg M, van der Velden R (2015). Skills and the graduate recruitment process: Evidence from two discrete choice experiments. Economics of Education Review.

[CR31] Hurst A (2019). Class and gender as predictors of study abroad participation among US liberal arts college students. Studies in Higher Education.

[CR32] Iriondo I (2020). Evaluation of the impact of Erasmus study mobility on salaries and employment of recent graduates in Spain. Studies in Higher Education.

[CR33] Jackson, J., Howard, M., & Schwieter, J. (2020). Language proficiency: Developmental perspectives and linguistic outcomes of education abroad. In A. Ogden, B. Streitwieser, & C. Van Mol (Eds.), *Education abroad: Bridging scholarship and practice* (pp. 92–105). Routledge.

[CR34] Jacob M, Kühhirt M, Rodrigues M (2019). Labour market returns to graduates’ international experience: Exploring cross-country variation in Europe. European Sociological Review.

[CR35] King R, Ruiz-Gelices E (2003). International student migration and the European ‘year abroad’: Effects on European identity and subsequent migration behaviour. International Journal of Population Geography.

[CR36] Kommers, S. (2020). *Are some horizons broader than others? Study abroad, inequality, and the influence on careers and education (Doctoral dissertation)*. University of Massachusetts. 10.7275/s3yw-z413.

[CR37] Kramer D, Wu J (2021). A HOPE for study abroad: Evidence from Tennessee on the impact of merit-aid policy adoption on study abroad participation. Educational Policy.

[CR38] Kratz F, Netz N (2018). Which mechanisms explain monetary returns to international student mobility?. Studies in Higher Education.

[CR39] Laurison D, Friedman S (2016). The class pay gap in higher professional and managerial occupations. American Sociological Review.

[CR40] Lingo M (2019). Stratification in study abroad participation after accounting for student intent. Research in Higher Education.

[CR41] Liwiński J (2019). Does studying abroad enhance employability?. Economics of Transition and Institutional Change.

[CR42] Liwiński J (2019). Does it pay to study abroad? Evidence from Poland. International Journal of Manpower.

[CR43] Lörz M, Netz N, Quast H (2016). Why do students from underprivileged families less often intend to study abroad?. Higher Education.

[CR44] Magnan S, Back M (2007). Social interaction and linguistic gain during study abroad. Foreign Language Annals.

[CR45] McKeown, J., Celaya, L., & Ward, H. (2020). Academic development: The impact of education abroad on students as learners. In A. Ogden, B. Streitwieser, & C. Van Mol (Eds.), *Education abroad: Bridging scholarship and practice* (pp. 77–91). Routledge.

[CR46] Medina-López-Portillo, A. (2004). Intercultural learning assessment: The link between program duration and the development of intercultural sensitivity. *Frontiers: The Interdisciplinary Journal of Study Abroad, 10*(1), 179–200. 10.36366/frontiers.v10i1.141.

[CR47] Messer D, Wolter S (2007). Are student exchange programs worth it?. Higher Education.

[CR48] Milstein T (2005). Transformation abroad: Sojourning and the perceived enhancement of self-efficacy. International Journal of Intercultural Relations.

[CR49] Ministerial Conference. (2009). The Bologna Process 2020 – The European Higher Education Area in the new decade. https://bit.ly/37CSvYX. Accessed on 7 July 2021.

[CR50] Ministerial Conference. (2012). Mobility for better learning. Mobility strategy 2020 for the European Higher Education Area. https://bit.ly/37ELwif. Accessed on 7 July 2021.

[CR51] Nerlich S (2021). Outcomes-focused evaluation of study abroad experiences. Journal of Higher Education Policy and Management.

[CR52] Netz N (2015). What deters students from studying abroad? Evidence from four European countries and its implications for higher education policy. Higher Education Policy.

[CR53] Netz, N., & Cordua, F. (2021). Does studying abroad influence graduates’ wages? A literature review. *Journal of International Students*, *11*(4). 10.32674/jis.v11i4.4008

[CR54] Netz N, Finger C (2016). New horizontal inequalities in German higher education? Social selectivity of studying abroad between 1991 and 2012. Sociology of Education.

[CR55] Netz, N., & Grüttner, M. (2020). Does the effect of studying abroad on labour income vary by graduates’ social origin? Evidence from Germany. *Higher Education*. 10.1007/s10734-020-00579-2.

[CR56] Netz, N., Klasik, D., Entrich, S., & Barker, M. (2020). Socio-demographics: A global overview of inequalities in education abroad participation. In A. Ogden, B. Streitwieser, & C. Van Mol (Eds.), *Education abroad: Bridging scholarship and practice* (pp. 28–42). Routledge.

[CR57] Netz, N., & Sarcletti, A. (2021). (Warum) beeinflusst ein Migrationshintergrund die Auslandsstudienabsicht? In M. Jungbauer-Gans & A. Gottburgsen (Eds.), *Migration, Mobilität und soziale Ungleichheit in der Hochschulbildung* (pp. 103–136). Springer Fachmedien. 10.1007/978-3-658-31694-5_5.

[CR58] Nguyen A-M, Jefferies J, Rojas B (2018). Short term, big impact? Changes in self-efficacy and cultural intelligence, and the adjustment of multicultural and monocultural students abroad. International Journal of Intercultural Relations.

[CR59] Niehoff E, Petersdotter L, Freund P (2017). International sojourn experience and personality development: Selection and socialization effects of studying abroad and the Big Five. Personality and Individual Differences.

[CR60] Norris, E., & Dwyer, M. (2005). Testing assumptions: The impact of two study abroad program models. *Frontiers: The Interdisciplinary Journal of Study Abroad, 11*(1), 121–142. 10.36366/frontiers.v11i1.154.

[CR61] Parey M, Waldinger F (2011). Studying abroad and the effect on international labour market mobility: Evidence from the introduction of ERASMUS. The Economic Journal.

[CR62] Paus, E., & Robinson, M. (2008). Increasing study abroad participation: The faculty makes the difference. *Frontiers: The Interdisciplinary Journal of Study Abroad, 17*(1), 33–49. 10.36366/frontiers.v17i1.243.

[CR63] Perna L, Orosz K, Jumakulov Z, Kishkentayeva M, Ashirbekov A (2015). Understanding the programmatic and contextual forces that influence participation in a government-sponsored international student-mobility program. Higher Education.

[CR64] Petersdotter L, Niehoff E, Freund P (2017). International experience makes a difference: Effects of studying abroad on students’ self-efficacy. Personality and Individual Differences.

[CR65] Petzold K (2017). Studying abroad as a sorting criterion in the recruitment process: A field experiment among German employers. Journal of Studies in International Education.

[CR66] Petzold K (2017). The role of international student mobility in hiring decisions. A vignette experiment among German employers. Journal of Education and Work.

[CR67] Petzold, K. (2020). Heterogeneous effects of graduates’ international mobility on employers’ hiring intentions—Experimental evidence from Germany. *Higher Education*. 10.1007/s10734-020-00524-3.

[CR68] Petzold, K., & Moog, P. (2018). What shapes the intention to study abroad? An experimental approach. *Higher Education*, *75*, 35-54. 10.1007/s10734-017-0119-z.

[CR69] Pimpa N (2003). The influence of family on Thai students’ choices of international education. International Journal of Educational Management.

[CR70] Presley A, Damron-Martinez D, Zhang L (2010). A study of business student choice to study abroad: A test of the theory of planned behavior. Journal of Teaching in International Business.

[CR71] Pungas E, Täht K, Realo A, Tammaru T (2015). Does ethnicity matter in intentions to study abroad? Analysis of high school students in Estonia. Journal of Ethnic and Migration Studies.

[CR72] Richter J, Zimmermann J, Neyer F, Kandler C (2021). Do sojourn effects on personality trait changes last? A five-year longitudinal study. European Journal of Personality.

[CR73] Rodrigues, M. (2013). *Does student mobility during higher education pay? Evidence from 16 European countries*. European Commission. 10.2788/95642.

[CR74] Rodríguez C, Bustillo R, Mariel P (2011). The determinants of international student mobility flows: An empirical study on the Erasmus programme. Higher Education.

[CR75] Roy A, Newman A, Ellenberger T, Pyman A (2019). Outcomes of international student mobility programs: A systematic review and agenda for future research. Studies in Higher Education.

[CR76] Salisbury M, Paulsen M, Pascarella E (2010). To see the world or stay at home: Applying an integrated student choice model to explore the gender gap in the intent to study abroad. Research in Higher Education.

[CR77] Sánchez C, Fornerino M, Zhang M (2006). Motivations and the intent to study abroad among U.S., French, and Chinese students. Journal of Teaching in International Business.

[CR78] Savage M, Egerton M (1997). Social mobility, individual ability and the inheritance of class inequality. Sociology.

[CR79] Schafer M, Wilkinson L, Ferraro K (2013). Childhood (mis)fortune, educational attainment, and adult health: Contingent benefits of a college degree?. Social Forces.

[CR80] Schmidt S, Pardo M (2017). The contribution of study abroad to human capital formation. The Journal of Higher Education.

[CR81] Schnepf S, Colagrossi M (2020). Is unequal uptake of Erasmus mobility really only due to students’ choices? The role of selection into universities and fields of study. Journal of European Social Policy.

[CR82] Schnusenberg O, de Jong P, Goel L (2012). Predicting study abroad intentions based on the theory of planned behavior. Decision Sciences Journal of Innovative Education.

[CR83] Sigalas E (2010). Cross-border mobility and European identity: The effectiveness of intergroup contact during the ERASMUS year abroad. European Union Politics.

[CR84] Simon J, Ainsworth J (2012). Race and socioeconomic status differences in study abroad participation: The role of habitus, social networks, and cultural capital. ISRN Education.

[CR85] Sorrenti G (2017). The Spanish or the German apartment? Study abroad and the acquisition of permanent skills. Economics of Education Review.

[CR86] Teichler, U. (2011). International dimensions of higher education and graduate employment. In J. Allen & R. van der Velden (Eds.), *The flexible professional in the knowledge society. New challenges for higher education* (pp. 177–197). Springer.

[CR87] Teichler U, Janson K (2007). The professional value of temporary study in another European country: Employment and work of former ERASMUS students. Journal of Studies in International Education.

[CR88] Triventi M (2013). The role of higher education stratification in the reproduction of social inequality in the labor market. Research in Social Stratification and Mobility.

[CR89] Van Mol, C. (2013). Intra-European student mobility and European identity: A successful marriage? Population, Space and Place, 19(2), 209–222*. *10.1002/psp.1752

[CR90] Van Mol C (2017). Do employers value international study and internships? A comparative analysis of 31 countries. Geoforum.

[CR91] Van Mol, C. (2021). Exploring explanations for the gender gap in study abroad: A case study of the Netherlands. *Higher Education*. 10.1007/s10734-020-00671-7.

[CR92] Van Mol, C., Caarls, K., & Souto-Otero, M. (2020). International student mobility and labour market outcomes: An investigation of the role of level of study, type of mobility, and international prestige hierarchies. *Higher Education*. 10.1007/s10734-020-00532-3.

[CR93] Van Mol C, Timmerman C (2014). Should I stay or should I go? An analysis of the determinants of intra-European student mobility. Population, Space and Place.

[CR94] Vögtle E, Windzio M (2016). Networks of international student mobility: Enlargement and consolidation of the European transnational education space?. Higher Education.

[CR95] Waibel S, Petzold K, Rüger H (2018). Occupational status benefits of studying abroad and the role of occupational specificity – A propensity score matching approach. Social Science Research.

[CR96] Waibel, S., Rüger, H., & Ette, A. (2020). *Who benefits? Heterogeneous effects of international student mobility on occupational attainment*. Universität Hamburg.

[CR97] Waibel S, Rüger H, Ette A, Sauer L (2017). Career consequences of transnational educational mobility: A systematic literature review. Educational Research Review.

[CR98] Walker, I. (2020). Chapter 6 - Heterogeneity in the returns to higher education. In S. Bradley & C. Green (Eds.), *The economics of education: A comprehensive overview* (2nd ed., pp. 75–90). Academic Press. 10.1016/B978-0-12-815391-8.00006-9.

[CR99] Waters J, Brooks R (2010). Accidental achievers? International higher education, class reproduction and privilege in the experiences of UK students overseas. British Journal of Sociology of Education.

[CR100] Whatley M (2017). Financing study abroad: An exploration of the influence of financial factors on student study abroad patterns. Journal of Studies in International Education.

[CR101] Whatley, M., & Canché, M. (2021). A robust estimation of the relationship between study abroad and academic outcomes among community college students. *Research in Higher Education*. 10.1007/s11162-021-09647-7.

[CR102] Wiers-Jenssen J (2008). Does higher education attained abroad lead to international jobs?. Journal of Studies in International Education.

[CR103] Wiers-Jenssen J (2011). Background and employability of mobile vs. non-mobile students. Tertiary Education and Management.

[CR104] Wiers-Jenssen, J., & Støren, L. (2020). International student mobility and the transition from higher education to work in Norway. *Higher Education*. 10.1007/s10734-020-00564-9.

[CR105] Wiers-Jenssen, J., Tillman, M., & Matherly, C. (2020). Employability: How education abroad impacts the transition to graduate employment. In A. Ogden, B. Streitwieser, & C. Van Mol (Eds.), *Education abroad: Bridging scholarship and practice* (pp. 135–149). Routledge.

[CR106] Wiers-Jenssen J, Try S (2005). Labour market outcomes of higher education undertaken abroad. Studies in Higher Education.

[CR107] Williams T (2005). Exploring the impact of study abroad on students’ intercultural communication skills: Adaptability and sensitivity. Journal of Studies in International Education.

[CR108] Willis R, Rosen S (1979). Education and self-selection. Journal of Political Economy.

[CR109] Winship C, Harding D (2008). A mechanism-based approach to the identification of age–period–cohort models. Sociological Methods & Research.

[CR110] Wolff F, Borzikowsky C (2018). Intercultural competence by international experiences? An investigation of the impact of educational stays abroad on intercultural competence and its facets. Journal of Cross-Cultural Psychology.

[CR111] Xie Y, Brand J, Jann B (2012). Estimating heterogeneous treatment effects with observational data. Sociological Methodology.

[CR112] Zimmermann, J., Greischel, H., & Jonkmann, K. (2020). The development of multicultural effectiveness in international student mobility. *Higher Education*. 10.1007/s10734-020-00509-2.

[CR113] Zimmermann J, Greischel H, Jonkmann K, Neyer F (2021). Growth all along the road? Personality development and international contacts of (in)experienced sojourners. European Journal of Personality.

[CR114] Zimmermann, J., & Neyer, F. (2013). Do we become a different person when hitting the road? Personality development of sojourners. *Journal of Personality and Social Psychology, 105*(3), 515–530. 10.1037/a0033019. 10.1037/a0033019 23773042

